# Extrapleural pneumonectomy or pleurectomy/decortication for malignant pleural mesothelioma

**DOI:** 10.1007/s11748-014-0389-7

**Published:** 2014-03-19

**Authors:** Seiki Hasegawa

**Affiliations:** Department of Thoracic Surgery, Hyogo College of Medicine, 1-1 Mukogawa-cho, Nishinomiya, 663-8501 Japan

**Keywords:** Malignant pleural mesothelioma, Surgery, Extrapleural pneumonectomy, Pleyrectomy/decortication, Multimodality treatment

## Abstract

Malignant pleural mesothelioma (MPM) is associated with a very poor prognosis. Unlike other solid tumors, any type of planned surgery for MPM would be cytoreductive rather than radical. There are two types of surgery for MPM. Extrapleural pneumonectomy (EPP) involves en bloc resection of the lung, pleura, pericardium, and diaphragm. Pleurectomy/decortication (P/D) is a lung-sparing surgery that removes only parietal/visceral pleura. In comparison with EPP, P/D is theoretically less radical but is associated with less perioperative mortality/morbidity and less postoperative deterioration of cardiopulmonary function. It still remains unclear which surgical technique is superior in terms of the risk/benefit ratio. In this context, selection between EPP and P/D has been a matter to debate.

## Introduction

Malignant pleural mesothelioma (MPM) is associated with a very poor prognosis, and its incidence is expected to increase in Asia and developing countries [1–6]. Because any type of planned surgery would be cytoreductive rather than radical [7], an optimal outcome via surgery alone is unlikely [8]. Accordingly, the current strategy for curing this disease has shifted to multimodal therapy with chemotherapy and/or radiation therapy (RT).

There are two types of surgery for MPM. Extrapleural pneumonectomy (EPP) involves en bloc resection of the lung, pleura, pericardium, and diaphragm. Pleurectomy/decortication (P/D) is a lung-sparing surgery that removes only parietal/visceral pleura. EPP leaves less residual tumor cells compared with P/D; however, it often results in high mortality/morbidity, severe depression of cardiorespiratory function, and poor quality of life. Till date, the risk–benefit ratios of P/D and EPP as part of multimodal therapy have not been clearly elucidated.

Furthermore, the decision to perform either EPP or P/D in studies on multimodal approaches has been solely based on surgical conjecture and bias, rather than scientific data [9].

## EPP and P/D surgical procedures

**Fig. 1:**
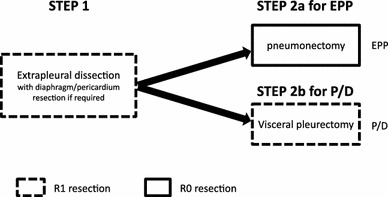
Diagram of surgical procedures in EPP and P/D. Step 1 comprises the common procedures in EPP and P/D, including thoracotomy, extrapleural dissection of the parietal pleura, with diaphragm and/or pericardium resection if required, and systematic lymph node dissection. Steps 2a and 2b represent other surgical options

The first set of procedures are common between EPP and P/D [10] (shown as Step 1 in Fig. 1). Step 1 involves thoracotomy, extrapleural dissection of the parietal pleura, with diaphragm and/or pericardium resection if required, and systematic lymph node dissection. Therefore, after completing step 1, the lung/pleura block is connected to the body only by hilar components, namely the main bronchus, main pulmonary artery, and pulmonary veins. The second set of procedures involve en bloc extirpation of lung, parietal/visceral pleura, diaphragm, and pericardium in EPP (Step 2a) and visceral pleurectomy in P/D (Step 2b).

Microscopic complete resection (R0) is theoretically impossible in Step 1 and Step 2b, but not in Step 2a. Step 2b is more likely to leave residual tumor cells compared with Step 1 because connection between the visceral pleura and lung parenchyma is usually tighter than that between the parietal pleura and chest wall. Therefore, P/D is less radical compared with EPP [11].

On the other hand, EPP has several disadvantages such as higher perioperative mortality/morbidity, severe deterioration of postoperative cardiopulmonary function and quality of life, and poorer tolerance to chemotherapy in case of recurrence.

**Fig. 2:**
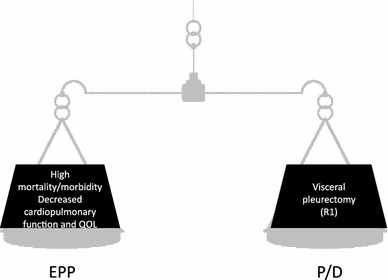
Comparison of disadvantages between EPP and P/D. EPP is associated with high perioperative mortality/morbidity and severe deterioration of postoperative cardiopulmonary function and quality of life. On the other hand, P/D leaves more residual tumor cells because of visceral pleurectomy. Selection between EPP and P/D ultimately leads to the selection of the radicality of Step 2a over that of Step 2b or the selection of less surgical insult from P/D over that from EPP

Therefore, selection between EPP and P/D leads to the selection of the radicality of Step 2a over that of Step 2b or the selection of less surgical insult from P/D over that from EPP (Fig. 2).

### Confusion and unanswered questions regarding MPM treatment

An element of extreme confusion exists with regard to MPM treatment, particularly surgery. The proposed reasons are mentioned below.

## Questionable survival benefit of surgery

Different surgical procedures with curative intent can exist for malignant disease, such as lobectomy and segmentectomy for primary lung cancer. However, the situation is quite different for MPM surgery. Unlike the goal of surgery for other solid tumors, the goal of MPM surgery is not radical resection but macroscopic complete resection (MCR) because of the lack of surgical margins [7, 12]. Recently, Treasure et al. [13] concluded from the Mesothelioma and Radical Surgery (MARS) feasibility study that radical surgery in the form of EPP within trimodality therapy offered no benefit. However, apt interpretation of the MARS study remains debatable [14–19].

## Why is survival after P/D equal to or even better than that after EPP

Theoretically, P/D is less radical than EPP, even though both are only cytoreductive procedures. However, most hospitals have reported equal or even better survival after P/D than after EPP [20–23]. In the context of multimodal therapy, Cao et al. [24], on the basis of their meta-analysis, concluded that selected patients who underwent extended P/D had lower perioperative morbidity and mortality with similar, if not superior, long-term survival compared with those who underwent EPP. Furthermore, Lucklatz and others [22] reported that P/D combined with postoperative adjuvant therapy provided better survival compared with EPP, irrespective of factors such as advanced disease or surgically less fit patients.

Other than nonprospective settings and patient selection bias, there may be several explanations for this contradiction.

First, EPP is associated with higher perioperative mortality/morbidity. Cao et al. conducted a systematic analysis and demonstrated that perioperative mortality (2.9 vs. 6.8 %, *p* = 0.02) and morbidity (27.9 vs. 62.0 %, *p* < 0.0001) were significantly lower for patients who underwent extended P/D than for those who underwent EPP [24]. Second, patients who undergo P/D have more opportunities for additional therapy after recurrence compared with patients who undergo EPP. Bolukbas et al. [25] found that additional chemotherapy after recurrence was acceptable in 64 % patients who initially underwent P/D and 25 % patients who initially underwent EPP. Accordingly, survival after recurrence was longer in patients who underwent P/D than in those who underwent EPP [15, 23]. Third, because of better cardiopulmonary reserve, patients who undergo P/D are more equipped to fend off postoperative nononcological disorders such as pneumonia and cardiac failure compared with those who undergo EPP.

Because there is no randomized study comparing EPP and P/D, it remains unclear whether postoperative survival in P/D patients is really equal to or better than that in EPP patients.

## Ambiguity surrounding the definition of P/D

Although P/D has been performed for more than 30 years, confusion still surrounds the actual meaning of pleurectomy/decortication. Recently, the International Mesothelioma Interest Group (IMIG), in collaboration with the International Association for the Study of Lung Cancer (IASLC), published a Consensus Report that classified pleurectomy into three categories according to surgical technique [26].

1. Extended P/D: parietal and visceral pleurectomy to remove all gross tumor, with resection of the diaphragm and/or pericardium.
2. P/D: parietal and visceral pleurectomy to remove all gross tumor, without resection of the diaphragm or pericardium.
3. Partial pleurectomy: partial removal of parietal and/or visceral pleura for diagnostic or palliative purposes, leaving gross tumor behind.

However, several critical points remain unclear.

First, does P/D allow part of the pleura to be left behind as long as it contains no macroscopic tumor? The consensus report does not mandate that P/D include 100 % visceral pleurectomy; it requires only MCR or complete resection of macroscopic tumors. The National Comprehensive Cancer Network (NCCN) guidelines clearly define P/D as complete removal of involved pleura and all gross tumor [27]. This distinction is particularly important in cases of early MPM, in which 100 % resection of almost intact visceral pleura is technically difficult. Second, the consensus states that resection of the diaphragm and/or pericardium is not mandatory in extended P/D; however, it should be performed if required. If so, what does P/D indicate? In cases involving the diaphragm and/or pericardium, pleurectomy without resection of the diaphragm and/or pericardium should be categorized as partial pleurectomy instead of P/D. I would propose that extended P/D and P/D be redefined as P/D, which involves parietal and visceral pleurectomy to remove all gross tumor, with resection of the diaphragm and/or pericardium if required. By changing the meaning of P/D in terms of diaphragm and/or pericardium involvement, a more comprehensible and consistent definition will be realized.

Third, does P/D allow the resection of pulmonary parenchyma? Lang-Lazdunski and colleagues [28] reported that 12 % (5/41) P/D patients required either lobectomy or segmentectomy. Also, an ongoing multicenter phase II study in Japan permits the resection of pulmonary parenchyma [29].

## Discrepancy among guidelines

The NCCN guidelines recommend surgical resection for patients with clinical stage I–III MPM who are medically fit for and can tolerate surgery [27]. The NCCN guidelines also recommend that P/D should be the first option for early disease (confined to the pleural envelope, no N2 lymph node involvement) with favorable histology (epithelioid).

In Europe, both the European Respiratory Society (ERS)/European Society of Thoracic Surgery (ESTS) [30] and British Thoracic Society (BTS) [31] guidelines state that the role of surgical resection in MPM is very uncertain and that radical surgery should only be performed in clinical trials, in specialized centers, and as part of a multimodal treatment plan. They also state that P/D should not be proposed with a curative intent. Italian guidelines recommend EPP to achieve adequate local control of MPM and P/D for patients with minimal, early-stage disease [32].

Therefore, discrepancies concerning performance practices and recommendations for P/D and EPP clearly exist. Furthermore, many MPM centers in Europe and some in North America and Japan are currently performing P/D with curative intent [20, 21, 28, 29, 33–37].

Should the surgical techniques for MPM ever be refined, the arrant inconsistencies cited above must be identified and resolved as soon as possible.

Very recently, the attendees of the 2012 International Mesothelioma Interest Group Congress agreed that the type of surgery (EPP or P/D), as long as it pertains to MCR, shall depend on clinical factors and the surgeon’s individual judgment and expertise [17]. This concept would seem to hold much promise.

## Scarcity of prospective clinical studies on P/D

With regard to EPP, one phase III study [13] and several phase II studies have been reported till date [38–42]. Therefore, the MCR completion rate and overall survival for intent-to-treat patients can be calculated.

With regard to P/D, however, there are few completed phase II studies [43, 44] and a few ongoing phase II studies [29, 45]. Rusch et al. [43] reported in their phase II study that MPM was resectable in 78 % (28/36) patients. However, they did not describe the MCR completion rate. An ongoing Japanese phase II study is designed to observe the feasibility of induction chemotherapy using pemetrexed plus cisplatin followed by P/D in patients with resectable MPM [29]. This study appears promising in that it will clarify the MCR completion rate as well as the conversion rate from P/D to EPP.

## RT after P/D

Unlike in EPP, external beam radiation therapy following P/D has been contraindicated because of possible damage to the preserved ipsilateral lung [30, 46, 47].

Very recently, however, a few authors reported successful RT after P/D. Minatel et al. administered 50 Gy of hemithoracic radiation with helical tomotherapy following radical P/D. This protocol resulted in a median survival time of 33 months, progression-free survival of 29 months, and a 3-year survival rate of 49 %, with no fatal toxicity. [48] There is an ongoing phase II study at Memorial Sloan-Kettering Cancer Center in which hemithoracic pleural intensity-modulated radiation therapy (IMRT; 50.4 Gy in 28 fractions) is administered after induction chemotherapy and P/D [45]; an interim analysis found that this protocol had an acceptable toxicity [49].

From these observations, one can speculate that the reintroduction of RT after P/D can result in better local control and longer postoperative survival.

## Selection between EPP and P/D

There exist some cases for which only one type of surgery is indicated. For example, patients with poor cardiopulmonary function are only fit for P/D. In patients with bulky and deep invasion to the pulmonary parenchyma, MCR can be achieved only by EPP. In the remaining cases, surgeons have to choose either EPP or P/D. Two different approaches are currently employed in patients with stage I–III resectable MPM who can tolerate aggressive surgery.

## Selection of surgery on an individual basis

Some surgeons recommend tailoring of the surgical procedure to intraoperative findings, with the ultimate goal of achieving MCR using the procedure with the least morbidity [9, 50]. These surgeons elect to perform P/D in patients with minimal disease [9, 51]. P/D is also recommended if essential mediastinal structures (e.g., aorta and vertebral bodies) are found to be involved at thoracotomy [52].

This approach is accepted by most MPM centers in North America and Japan, as well as by some European centers [17].

## Preference of P/D

Although European guidelines advise that P/D should not be proposed with a curative intent [30, 31], an increasing number of centers have abandoned EPP and consider P/D with a curative intent as their basic approach toward resectable MPM [28, 35, 37, 53]. The feasibility of P/D in patients with advanced MPM may be questionable. Friedberg and others reported an MCR rate of 97 % (37/38) and a median survival of 21 months in their series of radical pleurectomy with intraoperative photodynamic therapy for advanced MPM. On the basis of their results, they theorized that MCR could be achieved with radical pleurectomy in all MPM cases in which MCR could be achieved with EPP [53]. Bolukbas et al. [54] reported that an MCR rate of 61.9 %, a surgical mortality of 4.8 %, a median survival of 21 months, and a 5-year survival of 28 % were achieved in patients with stage III MPM treated by trimodality therapy with radical pleurectomy.

## Current approach to resectable MPM at Hyogo College of Medicine (Fig. 3)

As mentioned above, we are currently selecting the least invasive surgical procedures for achieving MCR. Therefore, surgery is initiated with the intention of performing P/D, with the exception of some cases with extensive invasion of MPM to the pulmonary parenchyma. Resection of the diaphragm and/or pericardium is performed only after all efforts to preserve them fail. Although an ongoing Japanese feasibility study permits the sparing of the visceral pleura as long as it does not contain macroscopic tumor [29], we remove all the parietal/visceral pleura irrespective of the presence of macroscopic lesions. Lung resection is frequently performed during P/D to achieve MCR and/or decrease air leakage.

**Fig. 3:**
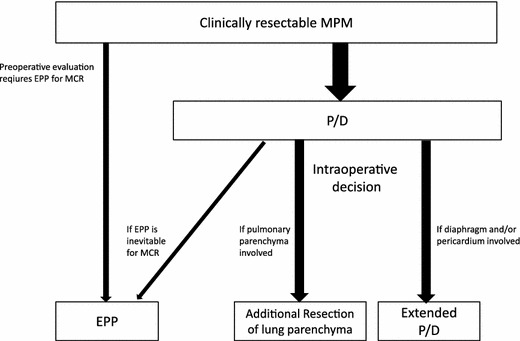
Current approach to resectable MPM at Hyogo College of Medicine. We are currently choosing the least invasive surgical procedures to achieve MCR. P/D is indicated in most cases, except those with extensive tumor invasion to the pulmonary parenchyma. Resection of the diaphragm, pericardium, and lung parenchyma is performed if required. Conversion to EPP from P/D is decided on the basis of intraoperative findings
